# Cortical and Deep Gray Matter Perfusion Associations With Physical and Cognitive Performance in Multiple Sclerosis Patients

**DOI:** 10.3389/fneur.2020.00700

**Published:** 2020-07-17

**Authors:** Dejan Jakimovski, Niels Bergsland, Michael G. Dwyer, John Traversone, Jesper Hagemeier, Tom A. Fuchs, Deepa P. Ramasamy, Bianca Weinstock-Guttman, Ralph H. B. Benedict, Robert Zivadinov

**Affiliations:** ^1^Department of Neurology, Buffalo Neuroimaging Analysis Center, Jacobs School of Medicine and Biomedical Sciences, University at Buffalo, State University of New York, Buffalo, NY, United States; ^2^IRCCS, Fondazione Don Carlo Gnocchi, Milan, Italy; ^3^Department of Neurology, Jacobs Multiple Sclerosis Center, Jacobs School of Medicine and Biomedical Sciences, University at Buffalo, The State University of New York, Buffalo, NY, United States; ^4^Center for Biomedical Imaging at Clinical Translational Science Institute, University at Buffalo, State University of New York, Buffalo, NY, United States

**Keywords:** MS, cerebral arterial blood flow, cognition, cardiovascular disease, hypertension, hyperlipidemia, heart disease, perfusion

## Abstract

**Background:** Reports suggest presence of cerebral hypoperfusion in multiple sclerosis (MS). Currently there are no studies that examine if the cerebral MS perfusion is affected by presence of cardiovascular comorbidities.

**Objective:** To investigate associations between cerebral perfusion and disease outcomes in MS patients with and without comorbid cardiovascular diseases (CVD).

**Materials:** One hundred three MS patients (75.7% female) with average age of 54.4 years and 21.1 years of disease duration underwent 3T MRI dynamic susceptibility contrast (DSC) imaging and were tested with Expanded Disability Status Scale, Multiple Sclerosis Severity Score (MSSS), Timed 25-Foot Walk (T25FW), 9-Hole Peg Test (9HPT) and Symbol Digit Modalities Test (SDMT). Structural and perfusion-based normalized measures of cerebral blood flow (nCBF), cerebral blood volume (nCBV) and mean transit time (MTT) of global, tissue-specific and deep gray matter (DGM) areas were derived. CBV and CBF were normalized by the normal-appearing white matter counterpart.

**Results:** In linear step-wise regression analysis, age- and sex-adjusted, MSSS (*R*^2^ = 0.186) was associated with whole brain volume (WBV) (β = −0.244, *p* = 0.046) and gray matter (GM) nCBF (β = −0.22, *p* = 0.035). T25FW (*R*^2^ = 0.278) was associated with WBV (β = −0.289, *p* = 0.012) and hippocampus nCBV (β = −0.225, *p* = 0.03). 9HPT (*R*^2^ = 0.401) was associated with WBV (β = 0.195, *p* = 0.049) and thalamus MTT (β = −0.198, *p*=0.032). After adjustment for years of education, SDMT (*R*^2^ = 0.412) was explained by T2-lesion volume (β = −0.305, *p* = 0.001), and GM nCBV (β = 0.236, *p* = 0.013). No differences in MTT, nCBF nor nCBV measures between patients with (*n* = 42) and without CVD (*n* = 61) were found. Perfusion-measures were also not able to distinguish CVD status in a logistic regression model.

**Conclusion:** Decreased GM and deep GM perfusion is associated with poorer MS outcomes, but not with presence of CVD.

## Introduction

Multiple sclerosis (MS) is a chronic inflammatory and demyelinating autoimmune disease of the central nervous system (CNS), characterized by episodes of neurological worsening, followed by either complete or partial restoration and concurrent neurodegeneration (1). Furthermore, varying proportion MS patients (from 35% in relapsing-remitting, up to 60% in secondary progressive MS) exhibit a significant cognitive impairment, particularly within the domain of cognitive processing speed (2). New and improved disease modifying treatments (DMTs) and greater access to specialized MS healthcare have led to a substantial increase in overall MS prevalence, as a result of decreased mortality rate while maintaining steady incidence rate (3). With an aging MS population, clinicians and researchers alike have raised concerns regarding an increased risk and frequency of vascular comorbidities like myocardial infarction, stroke, hypertension, heart failure, and abnormal lipid profiles when compared to healthy controls (4). Presence of these pathological vascular abnormalities have the potential to decrease overall quality of life, worsen cerebral perfusion and may additionally contribute to greater disease progression (5). Furthermore, patients affected by cerebrovascular diseases are associated with worse cognitive performance and MRI-derived MS outcomes (5).

To assess changes in cerebral perfusion and its implications on pathological MS processes, multiple studies have used various MRI methods including dynamic susceptibility contrast (DSC), dynamic contrast-enhanced (DCE) and arterial spin labeling (ASL) perfusion-weighted imaging (PWI) (6). As such, these methods can provide valuable quantitative measures like total cerebral blood volume (CBV) and cerebral blood flow (CBF) (6). The processes of MS lesion formation and repair are dependent on continues influx of inflammatory cells, successful debridement, and delivery of oxygenated blood. A large perfusion-based study showed discrepancies between greater lesion formation occurring in highly perfused white matter (WM) compared to greater accumulation of chronic MS lesion in physiologically hypoperfused areas (7). Moreover, perfusion measures are able to expose previously undetected widespread changes in the otherwise normal-appearing brain tissue and supplement the classically monitored MS lesions. In similar manner, hypoperfusion of the thalamus, a deep gray matter (DGM) region highly implicated in MS, has been associated with worse disability and composite clinical scores (8). Lastly, MS patients with cognitive impairment demonstrate greater cortical perfusion deficits despite any evidence of structural abnormalities or significant brain atrophy (9). The changes in DSC and ASL-based perfusion measures within the cortical and DGM in MS patients and their association with clinical outcomes have been recently reviewed elsewhere (6). These findings were supplemented by a study showing associations of decreased total cerebral extracranial blood inflow and lower cognitive performance (10). However, no MS studies have previously investigated changes in cerebral perfusion in regards to presence of comorbid cardiovascular diseases (CVD).

Based on this background, we hypothesized that presence of cerebral perfusion abnormalities within the GM are associated with MS-specific clinical and cognitive performance of a large sample of heterogeneous MS population, and that perfusion measures are associated with presence of CVD.

## Materials and Methods

### Study Population

Patients were recruited within a larger, prospective, longitudinal study that aimed to determine association between MRI and cardiovascular, genetic and environmental characteristics of MS patients (CEG-MS) (11, 12). The inclusion criteria for the MS patients were: (1) age between 18 and 75 years old, (2) being diagnosed with MS per 2010-revised McDonald criteria (13) or being clinically isolated syndrome (CIS) patients, (3) have MRI examination within 30 days of the clinical visit, (4) presence of PWI-based sequences in the MRI protocol, (5) availability of cognitive testing. Contrarily, MS patients were excluded if: (1) having clinically-defined relapse or have received intravenous corticosteroids within 30 days of the MRI examination, (2) pregnant or nursing mother, (3) having anatomical extracranial or intracranial vascular malformation like Parkes-Weber, Servelle-Martorell, Klippel-Trenaunay-Weber, or Budd-Chiari syndromes, (4) major depressive disorder or any other psychiatric comorbidities that can potentially influence cognitive performance and (5) presence of other major neurological disorders. An experienced neurologist examined the MS patients. Expanded Disability Status Scale (EDSS) and Multiple Sclerosis Severity Score (MSSS) were used to characterize disability levels (14, 15). Furthermore, timed 25-foot walk (T25FW) and 9-hole peg test (9HPT) were additionally administered (16, 17). Longer test times indicate worse walking and hand function, respectively. Symbol Digit Modalities Test (SDMT) determined cognitive processing speed performance, where higher raw scores indicated better performance (18). The MS phenotypes of relapsing-remitting MS (RRMS) and progressive MS (PMS) were determined based on medical history and clinical presentation as per 2013 Lublin criteria (19). Clinical information regarding disease duration, 5-year relapse rate, presence of CVD was collected by structured in-person questionnaire and further corroborated with electronic medical records, as previously reported (5, 10, 20). Diagnosis of heart disease, hypertension, diabetes, and hyperlipidemia and body-mass-index (BMI) were assessed. BMI ≥30 was considered as obesity. Lastly, the use of disease modifying treatment (DMT) was determined and categorized. All participants signed written consent form and local Institutional Review Board (IRB) approved the study.

### MRI Acquisition and Analysis

The MS patients underwent an MRI examination on a 3T Signa Excite HD 12 Twin-Speed scanner (GE, Milwaukee, WI, USA) equipped with 8-channel head and neck coil. Sequences/acquisitions used in this analysis included: (1) 3D spoiled-gradient recalled (SPGR) high-resolution T1w(weighted) sequence with echo time(TE)/inversion time(IR)/repetition time(TR) of 2.8/900/5.9 ms, flip angle (FLIP) of 10 degrees, voxel size of 1 × 1 × 1 mm^3^ and no gaps, (2) Fluid attenuated inversion recovery (FLAIR) sequence with TE/IR/TR of 120/2,100/8,500 ms, FLIP = 90 degrees and same voxel size of 1 × 1 × 1 mm^3^ with no gap, (3) Dynamic susceptibility contrast (DSC) was acquired with a single-shot echo-planar imaging sequence with TR/TE of 2,275/45 ms, FLIP = 90 degrees, echo train length = 1, bandwidth = 250 kHz, 1 × 1 × 4 mm^3^ without gap which was acquired during and after injection of during and after injection of 15 ml of 0.1 mM/kg gadolinium-diethylenetrimanie penta-acetic acid by power injector at speed of 5 ml/s. A total of 40 volumes were acquired.

T2 lesion volume (LV) was determined by an experienced clinical neuroimager (13 years of experience) using a validated semi-automated contouring/thresholding procedure conducted with Java Imaging Manipulation (JIM) software (Xinapse Systems, version 6.0, Essex, UK) on FLAIR sequence. Intra-rater agreement is evaluated annually with good agreement (ICC ≥ 0.8). Global volumes of whole brain (WBV; WBV = GMV + WMV + cerebellar volume), WM (WMV), gray matter and (GMV; GMV = cortical + deep gray matter) were derived by SIENAX software (FMIRB, Oxford, UK) and scaled for differences in skull size (by using the SIENAX scaling factor) (21). FMRIB's Integrated Registration and Segmentation Tool (FIRST) software derived the regional, nuclei-specific volumes of total DGM, thalamus, caudate, putamen, globus pallidus, and hippocampus. In order to prevent tissue misclassification, both global and nuclei specific volumes were derived after inpainting for T1 hypointense lesions which were semi-automatically segmented on the T1w sequence.

The PWI-derived measures of cerebral blood volume (CBV), cerebral blood flow (CBF), and mean transit time (MTT) were calculated with the JIM Perfusion tool. To obtain the arterial input function, we utilized the automatic arterial input (AIF) function detection feature, which searches for voxels that have AIF-like behavior. For all analyses, the selected voxels were visually inspected to ensure accurate identification.

By utilizing the first steady-state volume before contrast arrival, FMRIB's Linear Image Registration Tool (FLIRT) was used to bring other images into DSC space with a rigid body registration. Mean values for CBV, CBF, and MTT were calculated in the SIENAX and FIRST-based ROIs. (Supplement Figure 1). As an absolute quantification of CBV and CBF could not be performed due to technical limitations of the DSC sequence utilized, we normalized all derived perfusion parameters to the normal-appearing WM (NAWM) (22). On the other hand, MTT is an absolute measure and was quantified in seconds. Only the WBV was utilized as a structural volumetric measure in the models described hereafter.

### Statistical Analysis

The statistical analyses were performed using SPSS version 25.0 (IBM, Armonk, NY, USA). The data distribution was assessed by Kolmogorov-Smirnov test and by visual inspection of Q-Q plots. EDSS and MSSS were normalized by natural logarithmic transformation. For normalization of T25FW and 9HPT, two-step approach was utilized. After initial transformation of the data into fractional ranks, an inverse distribution function utilized the mean and standard deviation and returned the values into normal distribution where the cumulative probability is Prob(X < x). For demographic, clinical, and cognitive comparisons between the RRMS and PMS groups, χ^2^, Student's *t*-test, Mann-Whitney *U*-test, and age- and sex-adjusted analysis of covariance (ANCOVA) were used. Five linear regression models assessed associations between each specific clinical or cognitive outcomes with the MRI-derived measures. The models were constructed by initial block that adjusted for effects derived from age, sex, and years of education. The second block utilized step-wise hierarchical inclusion of scalar WBV and perfusion-based variables (independent) with an increasing explanatory power of respective outcome variance (dependent). Regression-based *R*^2^, standard error of the estimate, standardized β, and t-statistics were reported. *P* < 0.05 were considered statistically significant. The significant predictors in the models were also adjusted for multiple comparisons using false discovery rate (Benjamini-Hochberg procedure). Differences in MTT between MS patients with and without at least one of the aforementioned cardiovascular disease were determined by age-adjusted ANCOVA analysis. Logistic regression model determined MRI-based predictors of CVD presence. Lastly, the absolute values derived by automatic arterial input (AIF) are compared between patients with and without CVD.

## Results

### Demographic, Clinical, Cognitive, and MRI-Based Characteristics

Demographic, clinical and cognitive characteristics of the study population are shown in Table 1. A total of 103 MS patients was composed of 63 CIS/RRMS (6 CIS and 57 RRMS) and 40 PMS individuals and had an average age of 54.5 years, disease duration of 21.1 years, 5-year relapses rate of 0.155, median disability EDSS score of 3.0 and median MSSS score of 2.26. In particular, the median T25FW and 9HPT performance were 5.4 s and 23.4 s, respectively. Similarly, the average SDMT performance was 49.5. Use pattern of DMT is also shown in Table 1. Forty two MS patients (40.7%) had a diagnosis of at least one CVD. In particular, 18 (17.5%) MS patients had hypertension, 24 (23.3%) hyperlipidemia, 14 (13.6%) heart disease, 27 (26.2%) were obese, and 2 (1.9%) diabetes. As expected, the PMS population was older (Student's *t*-test, *p* < 0.001), had longer disease duration (Student's *t*-test, *p* < 0.001), had greater disability scores (Mann Whitney *U*-test, *p* < 0.001 for both EDSS and MSSS), had poorer walking, hand and cognitive processing speed performance (Mann-Whitney *U*-test, all *p* < 0.001) and had significantly lower 5-year relapse rate (Mann Whitney *U*-test, *p* = 0.008). PMS patients had numerically greater number of CVD and significantly higher percent of them were hypertensive (χ^2^, *p* = 0.001).

**Table 1:** Demographic, clinical, and cognitive characteristics of the study population.

**Demographic and clinical characteristics**	**MS** **(*n* = 103)**	**CIS+RRMS (*n* = 63)**	**PMS** **(*n* = 40)**	**RRMS vs. PMS *p*-value**
Female, *n* (%)	78 (75.7)	47 (74.6)	31 (77.5)	0.738
Age, mean (SD)	54.5 (11.7)	49.9 (11.8)	61.5 (7.3)	**<0.001**
Disease duration, mean (SD)	21.1 (10.9)	17.1 (9.6)	27.4 (9.9)	**<0.001**
EDSS, median (IQR)	3.0 (1.5–6.0)	2.0 (1.5–2.63)	6.5 (4.0–6.5)	**<0.001**
MSSS, median (IQR)	2.26 (1.0–5.6)	1.28 (0.78–2.49)	5.6 (3.4–6.7)	**<0.001**
BMI, mean (SD)	27.3 (5.3)	27.0 (5.3)	27.9 (5.4)	0.42
Obese, *n* (%)	27 (26.2)	13 (20.6)	14 (35.0)	0.115
At least one CVD, *n* (%)	42 (40.7)	22 (34.9)	20 (50.0)	0.129
Hypertension, *n* (%)	18 (17.5)	5 (7.9)	13 (32.5)	**0.001**
Hyperlipidemia, *n* (%)	24 (23.3)	12 (19.0)	12 (30.0)	0.2
Heart disease, *n* (%)	14 (13.6)	10 (15.9)	4 (10.0)	0.397
Diabetes, *n* (%)	2 (1.9)	0 (0)	2 (5.0)	**-**
5-year relapse rate, mean (SD)	0.155 (0.377)	0.232 (0.459)	0.031 (0.088)	**0.008**
T25FW, median (IQR)	5.4 (4.5–7.7)	4.8 (4.3–5.8)	7.7 (6.2–13.5)	**<0.001**
9HPT, median (IQR)	23.4 (19.9–30.4)	20.9 (18.9–24.5)	29.4 (24.5–41.3)	**<0.001**
SDMT, mean (SD)	49.5 (14.8)	53.8 (13.3)	42.6 (14.5)	**<0.001**
**DMT use**, ***n*** **(%)**				
Interferon-beta	31 (30.1)	23 (36.5)	8 (20)	0.742
Glatiramer acetate	28 (27.2)	15 (23.8)	13 (32.5)	
Natalizumab	3 (2.9)	2 (3.2)	1 (2.5)	
Oral medications	10 (9.7)	6 (9.5)	4 (10)	
Off-label medications	5 (4.9)	3 (4.8)	2 (5)	
No DMT use	26 (25.2)	14 (22.2)	12 (30)	

*MS, multiple sclerosis; CIS, clinically isolated syndrome; RRMS, relapsing remitting MS; PMS, progressive MS; EDSS, expanded disability status scale; MSSS, multiple sclerosis severity score; BMI, body mass index; T25FW, timed 25-foot walk; 9HPT, 9-hole peg test; SDMT, symbol digit modalities test; DMT, disease modifying treatment; CVD, cardiovascular disease; SD, standard deviation; IQR, interquartile range. Oral medications included dimethyl fumarate, teriflunomide, and fingolimod, whereas off-label medications included intravenous immunoglobulins, azathioprine, rituximab, mitoxantrone. Comparisons between the groups were performed by Student's t-test, χ^2^ and Mann-Whitney U-test, accordingly. P < 0.05 were considered statistically significant and shown in bold*.

The specific global, regional and lesion brain volumes of the study population are shown in Supplement Table 1.

### Associations Between Clinical, Cognitive, and Perfusion-Based Measures

The association between clinical scores, walking performance, hand performance and cognitive processing speed performance with MRI-derived measures are shown in Table 2. After adjustment for age and sex, greater EDSS scores were associated with smaller WBV (*R*^2^ increase from 32.5 to 40.9%, WBV standardized β = −0.345, *t*-statistics = −3.366, and *p* = 0.001). On the other hand, in addition to age and sex, the MSSS variance was additionally explained by GM nCBF (*R*^2^ increase of 4.0%, standardized β = −0.22, *t*-statistics = −2.14, *p* = 0.035) and WBV (*R*^2^ increase of 2.9%, standardized β = −0.244, *t*-statistics = −2.027, and *p* = 0.046). When WBV was introduced in the first initial correction regression step, the GM nCBF was retained in the step-wise model.

**Table 2:** Association between clinical MS scores (EDSS and MSSS) and MRI-derived measures.

**EDSS**	***R^**2**^***	**SE of estimate**	***t*-statistics**	**Standardized β**	***p*-value**
Block 1	0.325	0.55			
Sex	–	–	0.318	0.029	0.751
Patients' age	–	–	6.142	0.566	<0.001^*^
**Block 2**					
+WBV	0.409	0.52	−3.366	−0.345	0.001^*^
**MSSS**					
Block 1	0.115	0.92			
Sex	–	–	−0.499	−0.052	0.619
Patients' age	–	–	3.285	0.343	0.001^*^
**Block 2**					
+ GM nCBF	0.155	0.89	−2.14	−0.22	**0.035**
+ WBV	0.186	0.87	−2.027	−0.244	**0.046**
**T25FW**					
Block 1	0.161	20.6			
Sex	–	–	3.635	0.078	0.459
Patients' age	–	–	0.744	0.381	<0.001^*^
**Block 2**					
+ WBV	0.232	19.9	−2.582	−0.289	0.012^*^
+ Hippocampus nCBV	0.278	19.4	−2.214	−0.225	**0.03**
**9HPT**					
Block 1	0.333	20			
Sex	–	–	3.656	0.367	<0.001^*^
Patients' age	–	–	2.22	0.198	0.029^*^
**Block 2**					
+ Thalamus MTT	0.371	19.6	−1.992	−0.198	**0.032**
+ WBV	0.401	19.2	2.177	0.195	**0.049**
**SDMT**					
Block 1	0.276	12.6			
Sex	–	–	−0.831	−0.078	0.408
Patients' age	–	–	−5.068	−0.477	<0.001^*^
Years of education	–	–	1.992	0.186	0.05
**Block 2**					
+ T2 LV	0.364	11.9	−3.364	−0.305	0.001^*^
+ GM nCBV	0.412	11.5	2.546	0.236	0.013^*^

MS, multiple sclerosis; EDSS, Expanded Disability Status Scale; MSSS, Multiple Sclerosis Severity Score; T25FW, Timed 25-foot walk; 9HPT, 9-hole peg test; SDMT, Symbol Digit Modalities Test; WBV, whole brain volume; GM, gray matter; nCBF, normalized cerebral blood flow; nCBV, normalized cerebral blood volume; MTT, mean transit time; LV, lesion volume; SE, standardized error. Higher EDSS and MSSS score indicate greater disability. Longer T25FW time (in seconds) indicates worse walking ability. Longer 9HPT time (in seconds) indicates poorer hand performance. Higher SDMT score indicates better cognitive processing speed.

The regression models were constructed by initial block which force entered and adjusted for effects derived from age and sex (regardless of their significance). The second block utilized step-wise hierarchical inclusion of variables (independent) with an increasing explanatory power of respective outcome variance (dependent). Only significant predictors that provide additional power outside of block 1 are included in this block. In the case of SDMT model, years of education is additionally added in the first force entered block.

P < 0.05 were considered statistically significant and shown in bold.

* *- Survives false discovery rate (FDR) correction with Benjamini-Hochberg procedure*.

Age and sex explained a total of 16.1% of T25FW performance variance. Both WBV (*R*^2^ increase of 7.1%, standardized β = −0.289, *t*-statistics = −2.582, and *p* = 0.012) and hippocampus nCBV (additional *R*^2^ increase of 4.6%, standardized β = −0.225, *t*-statistics = −2.214, and *p* = 0.03) were able to further explain greater variance.

In a similar manner, age and sex were able to explain 33.6% of hand dexterity (9HPT) performance variance. Two additional variables were included in the step-wise hierarchical modeling. First, the thalamus MTT provided an additional 3.8% *R*^2^ increase (standardized β = −0.198, *t*-statistics = −1.992, *p* = 0.032). Furthermore, WBV explained 3.0% more *R*^2^ variance (standardized β = 0.195, *t*-statistics = 2.177, *p* = 0.049). Similarly to the MSSS analysis, when the WBV variable was in the first block of controlling variables, the thalamus MTT was also retained in the model.

**Figure 1:**
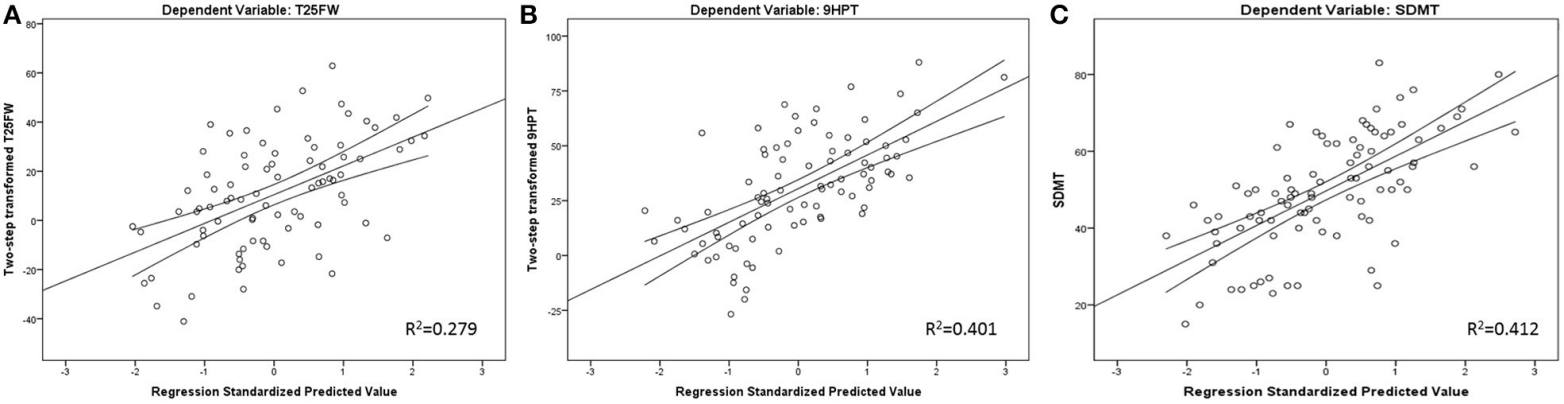
Regression-based plots for explained T25FW **(A)**, 9HPT **(B)**, and SDMT **(C)** variance. Legend: 9HPT, 9 Hole peg test; T25FW, Timed 25 foot walk; SDMT, Symbol Digit Modalities Test. Both 9HPT and T25FW were normalized by fractional ranks and an inverse distribution function.

Finally, 27.6% of cognitive processing speed performance variance was initially explained by sex, age, and years of education alone. Further *R*^2^ increases were noted by adding T2-LV (*R*^2^ increase of 8.8%, standardized β = −0.305, *t*-statistics = −3.364, *p* = 0.001) and GM nCBV (*R*^2^ increase of 4.8%, standardized β = 0.236, *t*-statistics = 2.546, *p* = 0.013). GM nCBV was the only significant perfusion-based predictor after multiple comparison correction for all regression models. Scatter plot representation for the final regression-based prediction models for 9HPT, T25FW, and SDMT are shown in Figure 1.

### Cardiovascular Diseases and Differences in Perfusion Measures

The demographic and clinical characteristics of MS patients with and without at least one CVD diagnosis are shown in Table 3. Although the CVD+ group had numerically more PMS patients (47.6 vs. 32.8%), this did not reach statistical significance (χ^2^, *p* = 0.357). In terms of perfusion-based measures, there were no significant differences between the MS patients with or without at least one CVD. Apart from age, presence of CVD was not predicted by any DSC measure in a step-wise logistic regression model [age Exp(B) = 1.087, *p* = 0.008]. Exploratory analysis of the logistic regression model showed that higher thalamic MTT was the only trending variable (stand-alone main effect of higher thalamic MTT values and presence of CVD with *p* = 0.052) Lastly, comparison of raw CBV and CBF (i.e., not normalized to NAWM) values were compared between MS patients with and without CVD and are shown in Supplement Table 2.

**Table 3:** Differences in MTT between MS patients with and without at least one CVD.

**Demographic and clinical characteristics**	**MS CVD + (*n* = 42)**	**MS CVD – (*n* = 61)**	**CVD + vs. CVD – *p*-value**
Female, *n* (%)	32 (76.2)	46 (75.4)	0.928
Age, mean (SD)	58.7 (9.3)	51.5 (5.4)	**0.002**
BMI, mean (SD)	28.5 (5.1)	26.6 (5.4)	0.08
Obese, *n* (%)	16 (38.1)	11 (18.0)	**0.039**
CIS/RRMS/PMS	2/20/20	4/37/20	0.357
Disease duration, mean (SD)	23.5 (11.1)	19.4 (10.5)	0.065
EDSS, median (IQR)	3.25 (1.88–6.5)	2.0 (1.5–6.0)	0.177
MSSS, median (IQR)	2.28 (1.28–6.0)	2.23 (0.92–5.1)	0.408
5-year relapse rate, mean (SD)	0.113 (0.364)	0.184 (0.385)	0.356
T25FW, median (IQR)	5.7 (4.6–8.2)	5.2 (4.5–6.9)	0.343
9HPT, median (IQR)	25.2 (21.7–32.7)	21.6 (19.1–29.1)	**0.028**
SDMT, mean (SD)	48.1 (14.8)	50.5 (14.8)	0.44
**DSC measures, mean (SD)**	**Age-adjusted ANCOVA**		
NABT MTT	3.47 (0.7)	3.44 (0.7)	0.802
NAWM MTT	3.52 (0.7)	3.5 (0.7)	0.836
GM MTT	3.44 (0.8)	3.42 (0.7)	0.721
DGM MTT	3.3 (0.8)	3.21 (0.7)	0.903
Thalamus MTT	3.25 (0.9)	3.1 (0.8)	0.768
GM nCBV	1.79 (0.15)	1.8 (0.25)	0.725
DGM nCBV	1.57 (0.1)	1.56 (0.17)	0.566
Thalamus nCBV	1.57 (0.16)	1.54 (0.19)	0.482
GM nCBF	1.95 (0.35)	1.9 (0.28)	0.209
DGM nCBF	1.89 (0.38)	1.83 (0.26)	0.258
Thalamus nCBF	1.72 (0.29)	1.71 (0.29)	0.414

MS, multiple sclerosis; CVD, cardiovascular disease; BMI, body mass index; MTT, mean transit time; nCBV, normalized cerebral blood volume; nCBF, normalized cerebral blood flow; EDSS, Expanded Disability Status Scale; MSSS, Multiple Sclerosis Severity Score; T25FW, timed 25-foot walk; 9HPT, 9-hole peg test; SDMT, Symbol Digit Modalities Test; NABT, normal-appearing brain tissue; NAWM, normal-appearing white matter; GM, gray matter; DGM, deep gray matter.

*Comparison between the MS patients with (CVD +) and without (CVD –) at least one CVD disease were done by Student's t-test, χ^2^ and Mann-Whitney U-test, accordingly. Analysis of covariance (ANCOVA) was used to compare perfusion-based MTT/nCBV/nCBV measures. Normalized CBV and CBF are calculated as a ratio of the respective measure in NAWM. P < 0.05 were considered statistically significant and shown in bold*.

## Discussion

In this study, we demonstrated significant associations between clinical and cognitive processing speed performance of MS patients with decreased cerebral perfusion as measured by DSC-PWI. These associations were independent of T2-LV and WBV effects and explained additional physical and cognitive disability variance. There were no differences in PWI-derived MTT between patients with at least one CVD when compared to patients without cardiovascular comorbidities.

Previous MS perfusion studies have reported mixed results with most studies demonstrating cerebral hypoperfusion and its effect on MS outcomes. As such, our results fall in line with the majority of the literature, which highlights the importance of cortical perfusion and its effect on MS outcomes including physical disability, cognitive impairment, and fatigue. For example, a small study of 22 MS patients and 11 age- and sex-matched healthy controls demonstrated associations between lower DGM CBV and CBF measures with patient-reported fatigue severity (23). Another recent MS voxel-based morphometry report demonstrated presence of significant reduction of both GM and DGM perfusion despite the lack of structural pathology (24). In addition, hypoperfusion was associated with impaired cognitive processing speed and lower performance on memory tests (24, 25). On the contrary, few studies were not able to replicate the perfusion findings reported in the literature finding lack of associations (26). A recent comprehensive summary of all previous perfusion studies and their results has been published elsewhere (4).

The importance of sufficient cerebral perfusion in maintaining proper cognitive functioning is recently highlighted in both cross-sectional and longitudinal studies of otherwise cognitively intact healthy population. For an example, a recent 2-year long longitudinal ASL study showed that healthy individuals with decline in GM CBF present with concurrent decline in other markers of brain aging including lower cortical volume, worse WM microstructural integrity, and greater appearance of WM hyperintensities (27). More importantly, the longitudinal GM CBF decline was associated with worsening of the cognitive processing speed (27). Reduction in cerebral perfusion has been also hypothesized as early marker of multiple neurodegenerative diseases, greater cerebral protein accumulation, and as a predictor of dementia development (28). As such, a population-based study has demonstrated that lower cerebral perfusion is associated with 31% greater risk of Alzheimer's disease development and accelerated decline in cognition (29). Lastly, presence of cardiovascular and cardiovascular-driven inflammation can expedite the cerebral vascular control and lead to greater cognitive decline (30). Given that MS patients present with greater prevalence of cardiovascular comorbidities when compared to the general population, lifestyle-based modification and appropriate CVD treatment may alleviate some of the perfusion-driven cerebral pathology (20). However, in our sample of 103 MS patients, the presence of CVD did not influence the MRI-derived perfusion measure. These findings may be explained by the low sample size and relatively low prevalence of CVDs. Furthermore, the CVD effect on MS perfusion may be more significant within an older MS population that presents with greater number of CVD comorbidities (3). Moreover, potential changes in cerebral perfusion due to persistent chronic NAWM inflammation and/or significant cortical lesions activity may be more prevalent within active MS population which is generally younger and have lower prevalence of CVD diagnoses. Lastly, since the recent hypertension guidelines have lowered the threshold to >130/80 mmHg, some hypertension-perfusion interactions may be present within our non-CVD MS population (31). Future studies should aim at determining whether the MS cerebral hypoperfusion has primary or secondary etiology, its relationship with cardiovascular comorbidities and a potential additive effect on disability accrual.

In a previous work, we demonstrated that cognitively impaired MS patients present with lower total extracranial arterial blood flow (10). The smaller amount of blood entering the cranium may be directly associated with lower cerebral perfusion measures including lower CBV, CBF, and prolonged MTT. In order to compensate for the variable extracranial blood flow, the neurovascular unit is responsible for adjusting the overall CBV by triggering vasodilatatory or vasoconstrictory responses. However, recent reports suggest that MS patients present with impaired functional cerebrovascular reactivity where they are not able to compensate during hypercapnic stimuli and result with neurodegenerative changes and brain atrophy (32). Therefore, the relative inability of the cerebral circulation to adapt will result in greater dependence on the cardiac output and on systematic arterial pressure to deliver the necessary brain blood flow. Presence of cardiovascular comorbidities will further exacerbate the extracranial arterial pathology and may result with even lower blood output to the brain (33). Contrarily, intact cerebrovascular reactivity processes can sufficiently compensate for early CVD-based hypoperfusion and attempt at maintaining sufficient blood flow. Therefore, future studies should aim at assessing the multifaceted nature of cerebral perfusion control and the consequences when one or more processes start to fail.

The study presents with several limitations. The perfusion measures derived from DSC are typically relative in nature and specific to the patient's hematocrit and relaxivity of the contrast agent (6). More advanced acquisitions and post-processing can be utilized to account for such factors, but were unavailable in the present study. Although significantly affected by partial volume effects, we utilized deconvolution with arterial input function and compared the absolute CBV and CBF (6). Moreover, the normalization of r CBV and rCBF with the respective NAWM perfusion rates can be influenced by varying rate of NAWM pathology. Furthermore, MS may present with spatially discrepant GM/WM pathology ratio, where some patients exhibit significantly more affected WM and other exhibit greater GM pathology. Such examples of exclusive myelocortical pathology distribution have been recently described (34). MS patients also demonstrate variable and undetectable NAWM pathology which can alter NAWM perfusion and in this case significantly influence the nGM CBF calculation. Studies can utilize MRI sequences that are able to provide absolute blood measure quantifications like ASL or book-end DSC method together with sequences that allow GM lesion quantification. The significant variability within the physiological brain perfusion can be attributed to more than 58 different perfusion modifiers divided into four large groups (physiological, blood components, mental state and caffeine/recreational drugs) (35). In particular controlling for age, blood gases and caffeine consumption can significantly decrease the inconsistency in the perfusion literature (35). Another limitation is the lack of healthy control population. A longitudinal, case-controlled design would provide better understanding of the temporal associations between changes in perfusion and their effect on clinical or cognitive outcomes.

In conclusion, decreased GM and DGM perfusion is independently associated with poorer clinical and cognitive outcomes in MS patients. These findings further corroborate the concurrent GM dependence of physical and cognitive functioning. Presence of cardiovascular comorbidity did not affect the perfusion-based MTT measure in this particular MS population.

## Data Availability Statement

All datasets generated for this study are included in the article/supplementary material. The data is further available from the corresponding author upon reasonable request.

## Ethics Statement

The studies involving human participants were reviewed and approved by University at Buffalo IRB. The patients/participants provided their written informed consent to participate in this study.

## Author Contributions

DJ: study concept and design, analysis and interpretation, drafting of the manuscript, critical revision of the manuscript for important intellectual content, and study supervision. RZ: study concept and design, analysis and interpretation, critical revision of the manuscript for important intellectual content, and study supervision. RB and BW-G: analysis and interpretation, critical revision of the manuscript for important intellectual content, and study supervision. DR, TF, JT, MD, NB, and JH: analysis and interpretation, critical revision of the manuscript for important intellectual content. All authors contributed to the article and approved the submitted version.

## Conflict of Interest

MD has received research grant support from Novartis and Keystone Heart. RB received personal compensation from Veraci, Genentech, Roche, Novartis, Genzyme, Sanofi and Biogen, Bristol Myers Squibb and EMD Serono for speaking and consultant fees. He received financial support for research activities from Genzyme, Genentech, Biogen, Verasci, and Mallinckrodt. BW-G received honoraria as a speaker and as a consultant for Biogen Idec, Teva Pharmaceuticals, EMD Serono, Novartis, Genentech and Mallickrodt BW-G received research funds from Biogen Idec, Teva Pharmaceuticals, EMD Serono, Novartis, Genentech and Mallinckrodt. RZ received personal compensation from EMD Serono, Genzyme-Sanofi, Celgene and Novartis for speaking and consultant fees. He received financial support for research activities from Genzyme-Sanofi, Novartis, Celgene, Mapi Pharma, Protembis, V-Wave Medical, and Keystone Heart. The remaining authors declare that the research was conducted in the absence of any commercial or financial relationships that could be construed as a potential conflict of interest.
