# Supplementary material for: Cortical and Deep Gray Matter Perfusion Associations With Physical and Cognitive Performance in Multiple Sclerosis Patients
Source: Front Neurol. 2020 Jul 17;11:700. doi: 10.3389/fneur.2020.00700 (PMC7380109; doi:10.3389/fneur.2020.00700)
Supplement: Supplementary file 1 [file Data_Sheet_1.docx]

**Supplement Material**

**Supplement Table 1.** MRI-derived volumetric measures of the study population.

| Global and regional volumes | MS  (n=103) | RRMS (n=63) | PMS  (n=40) | p-value |
| --- | --- | --- | --- | --- |
| T2-LV | 14.9 (18.9) | 9.7 (13.9) | 23.3 (22.5) | **0.01** |
| WBV | 1450.1 (95.9) | 1484.9 (85.6) | 1397.5 (87.0) | **0.016** |
| GMV | 735.7 (64.8) | 757.6 (61.2) | 702.6 (55.9) | 0.132 |
| WMV | 714.4 (44.2) | 727.3 (41.0) | 694.9 (42.0) | **0.005** |
| CV | 598.1 (51.7) | 614.3 (48.8) | 573.6 (46.6) | 0.249 |
| DGM | 54.3 (7.1) | 56.6 (6.1) | 57.4 (7.0) | **0.021** |
| Thalamus | 17.8 (2.5) | 18.7 (2.2) | 16.6 (2.5) | **0.025** |
| Caudate | 8.1 (1.3) | 8.5 (1.2) | 7.5 (1.2) | **0.023** |
| Putamen | 11.7 (1.6) | 12.2 (1.5) | 1.1 (1.6) | **0.027** |
| Globus pallidus | 3.9 (0.8) | 4.1 (0.8) | 3.8 (0.8) | 0.387 |
| Hippocampus | 8.8 (1.4) | 9.1 (1.3) | 8.2 (1.4) | 0.158 |

**Legend:** MS – multiple sclerosis, RRMS – relapsing-remitting MS, PMS – progressive MS, LV – lesion volume, WBV – whole brain volume, GMV – gray matter volume, WMV – white matter volume, CV – cortical volume, DGM – deep gray matter.

All MRI-derived metrics are shown as mean (standard deviation) and in milliliters (mL).

Differences in global and regional volumes were derived with age-adjusted analysis of covariance (ANCOVA). P-values lower than 0.05 were considered statistically significant.

**Supplement Table 2.** Comparison of absolute CBF and CBV DSC-based perfusion data between MS patients with and without CVD.

| DSC measures, mean (SD) | MS CVD +  (n=42) | MS CVD -  (n=61) | CVD + vs. CVD -  p-value |
| --- | --- | --- | --- |
| NAWB CBV | 0.276 (0.07) | 0.265 (0.07) | 0.26 |
| NAWM CBV | 0.202 (0.05) | 0.196 (0.06) | 0.569 |
| GM CBV | 0.359 (0.09) | 0.343 (0.09) | 0.167 |
| DGM CBV | 0.318 (0.09) | 0.296 (0.08) | 0.169 |
| Thalamic CBV | 0.321 (0.09) | 0.295 (0.09) | 0.165 |
| NAWB CBF | 561.6 (337.7) | 500.4 (204.1) | 0.083 |
| NAWM CBF | 378.8 (177.9) | 350.1 (149.2) | 0.169 |
| GM CBF | 753.5 (549.2) | 663.9 (267.4) | 0.074 |
| DGM CBF | 740.3 (432.3) | 632.6 (272.5) | 0.058 |
| Thalamic CBF | 678.4 (423.1) | 592.6 (272.9) | 0.089 |

**Legend:** MS – multiple sclerosis, CVD – cardiovascular disease, DSC – dynamic susceptibility contrast, CBV – cerebral blood volume, CBF – cerebral blood flow; NAWB – normal-appearing whole brain; NAWM – normal-appearing white matter; GM – gray matter; DGM – deep gray matter

Age-adjusted analysis of covariance (ANCOVA) was used. The data is derived using JIM software (version 6.0).

**Supplement Figure 1.** Deep gray matter nuclei segmentation


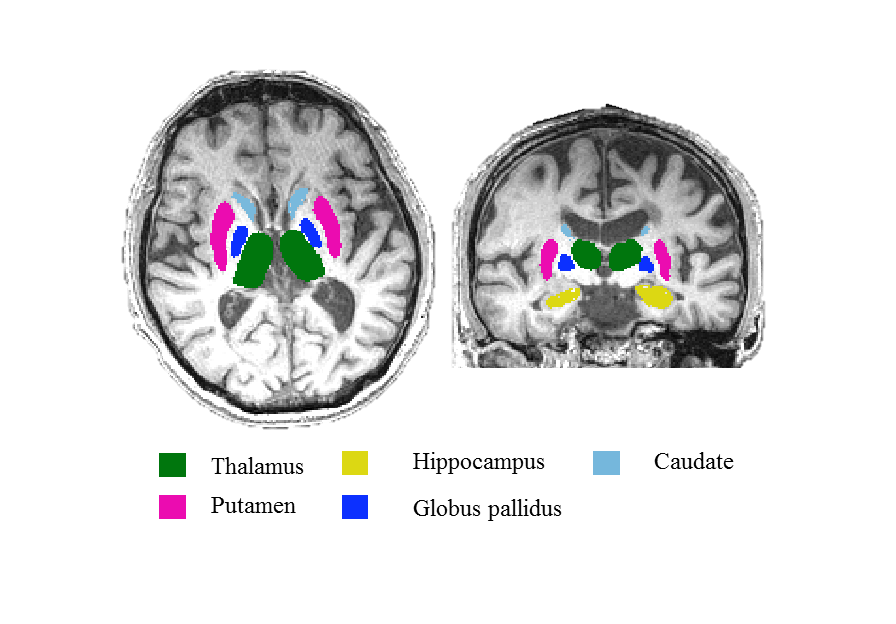
**Legend:** FIRST **-** FMRIB’s Integrated Registration and Segmentation Tool was used to determine the region of interest regarding the deep gray matter nuclei.
